# Circulating Cell-Free Nucleic Acids as Epigenetic Biomarkers in Precision Medicine

**DOI:** 10.3389/fgene.2020.00844

**Published:** 2020-08-11

**Authors:** Beenish Rahat, Taqveema Ali, Divika Sapehia, Aatish Mahajan, Jyotdeep Kaur

**Affiliations:** ^1^National Institute of Child Health and Human Development, National Institutes of Health, Bethesda, MD, United States; ^2^Postgraduate Institute of Medical Education and Research, Chandigarh, India

**Keywords:** autoimmune diseases, cancer diagnosis, precision medicine, epigenetic biomarkers, circulating nucleic acids in plasma/serum, prenatal and genetic diagnostics, circulating cell free nucleic acids

## Abstract

The circulating cell-free nucleic acids (ccfNAs) are a mixture of single- or double-stranded nucleic acids, released into the blood plasma/serum by different tissues via apoptosis, necrosis, and secretions. Under healthy conditions, ccfNAs originate from the hematopoietic system, whereas under various clinical scenarios, the concomitant tissues release ccfNAs into the bloodstream. These ccfNAs include DNA, RNA, microRNA (miRNA), long non-coding RNA (lncRNA), fetal DNA/RNA, and mitochondrial DNA/RNA, and act as potential biomarkers in various clinical conditions. These are associated with different epigenetic modifications, which show disease-related variations and so finding their role as epigenetic biomarkers in clinical settings. This field has recently emerged as the latest advance in precision medicine because of its clinical relevance in diagnostic, prognostic, and predictive values. DNA methylation detected in ccfDNA has been widely used in personalized clinical diagnosis; furthermore, there is also the emerging role of ccfRNAs like miRNA and lncRNA as epigenetic biomarkers. This review focuses on the novel approaches for exploring ccfNAs as epigenetic biomarkers in personalized clinical diagnosis and prognosis, their potential as therapeutic targets and disease progression monitors, and reveals the tremendous potential that epigenetic biomarkers present to improve precision medicine. We explore the latest techniques for both quantitative and qualitative detection of epigenetic modifications in ccfNAs. The data on epigenetic modifications on ccfNAs are complex and often milieu-specific posing challenges for its understanding. Artificial intelligence and deep networks are the novel approaches for decoding complex data and providing insight into the decision-making in precision medicine.

## Introduction

The diagnostic platform utilizing the detection of biomarkers in various body fluids called “liquid biopsy” can revolutionize precision medicine. Precision medicine is aimed at attaining better-personalized care by the development of the latest diagnostic and prognostic methods that consider individual variability (Kaur et al., 2017). Liquid biopsy is being utilized for non-invasive prognostic and predictive purposes. Efficient and reliable markers within the body fluids can help in personalized treatment decisions for monitoring disease and survival. ccfNAs have emerged as such markers for screening, diagnosis, prognosis, management, and treatment of various cancers; autoimmune, neurological, and mitochondrial diseases; prenatal diagnosis; diagnosis of pregnancy-related complications (Pos et al., 2018); diabetes; inflammation; rheumatoid arthritis; stroke; and trauma (Swarup and Rajeswari, 2007). An increased amount of ccfNAs is observed in these disorders, making liquid biopsies more sensitive, rapid, accurate, and preferable alternatives for various invasive diagnostic methods (Pos et al., 2018).

ccfNAs present in blood circulation include cell-free genomic DNAs (ccfDNAs) and cell-free mtDNA (Kohler et al., 2009; Thierry et al., 2016) and cell-free RNAs (ccfRNAs) including protein-coding messenger RNA (mRNA), regulatory non-coding RNAs like microRNAs (miRNAs), long non-coding RNAs (lncRNAs), circular RNAs, and RNAs involved in translation like transfer RNAs (tRNAs) and ribosomal RNAs (rRNAs) (Pos et al., 2018).

The ccfNAs (DNAs and RNAs) are generally released into the blood circulation either by apoptosis, necrosis, or active secretion. In healthy persons, the origin of ccfNAs is mainly attributed to lymphoid and myeloid tissues (Snyder et al., 2016), while in the case of various clinical conditions, the associated or the affected tissues would release the extra amount of ccfNAs into blood (Swarup and Rajeswari, 2007; Devonshire et al., 2014) in a pattern specific to the pathophysiological condition (Hunter et al., 2008; Noferesti et al., 2015).

Various genetic as well as epigenetic biomarkers have been explored for ccfNA-based liquid biopsy. As genetic biomarkers are less consistent and provide more variability across studies, epigenetic markers, which are more generalized between samples, present as a promising alternative for early diagnosis and monitoring of the diseases. These epigenetic marks are tissue specific and reflect the pattern of disease progression (Zeng et al., 2019). Furthermore, epigenetic biomarkers are dynamic with most techniques required for analysis of these biomarkers that are already available in clinical laboratories.

The use of epigenetic marks has revolutionized the field of non-invasive molecular diagnosis replacing traditional screening and treatment methods. These assays have great potential in future precise patient care. The epigenetic marks for ccfNAs reflect the pattern specific for the tissue contributing to these ccfNAs. Therefore, the use of epigenetic markers can help in the diagnosis of various diseases even before the onset of actual symptoms and hence help in better management of the disease. Precision medicine has improved health care by the identification of different stages/subsets of diseases, precise diagnosis, and treatment. Furthermore, the development of advanced analytical software techniques like machine learning and artificial intelligence can enhance precision medicine (Ahlquist, 2018; Beltran-Garcia et al., 2019). These are used in combination with next-generation sequencing to identify novel ccfNA-based epigenetic markers.

## Epigenetic Biomarkers in ccfNAs

Reliable markers are required to guide personalized treatment decisions for monitoring disease progression and survival. The presence of epigenetic marks on ccfNAs specific to a particular clinical condition is widely being explored to advance personalized medicine. A perfect epigenetic marker for precision medicine should be able to detect the disease with high sensitivity, predict the risk of disease development and its progression, and monitor the therapeutic response of the patient (Beltran-Garcia et al., 2019). ccfDNAs are associated with various epigenetic marks (Schwarzenbach et al., 2011) like DNA methylation, hydroxymethylcytosine (5hmC), and posttranslational modifications of histones. In addition, nucleosome positioning and occupancy on ccfDNAs have exhibited high sensitivity and specificity in liquid biopsy-based methods for disease detection and classification.

The 5-methylcytosine (5mC) modification at CpG dinucleotides is the most abundant form of DNA methylation. It plays an important role in the regulation of gene expression and is widely used as an epigenetic biomarker for ccfDNA-based assays. DNA methylation has replaced many genetic mutation- or protein-based markers. These 5mC biomarkers are also valuable in identifying tissue-specific methylation to estimate tumor burden and tissue of origin in ccfDNAs. In addition to 5mC, 5-hydroxymethylcytosine (5hmC) is also used as an epigenetic mark on ccfDNAs (Zeng et al., 2019). 5hmC is created by the oxidation of 5mC by 10–11 translocation (Tet) proteins. Although 5hmC is far less abundant compared to 5mC, it is more distinctly distributed among different transcriptionally active regions, which emphasizes its potential as a diagnostic marker. Genome-wide analysis of 5hmC pattern can provide more information about the potential of this epigenetic marker for ccfDNAs (Zeng et al., 2019).

Nucleosome positioning has emerged as a recent biomarker to distinguish the tissue of origin of ccfDNA based on derived nucleosome maps. Snyder et al., 2016 performed deep sequencing on ccfDNAs and observed a distinct pattern of nucleosome positioning between healthy persons and cancer patients correlating with the tissues of origin (Snyder et al., 2016). This emphasizes the use of nucleosome maps, which consist of occupancy of transcription factor and nucleosome as the epigenetic marks to distinguish normal versus cancer ccfDNAs. Hence, nucleosome positioning can also be used to identify various cancers that generally require invasive biopsies for definitive diagnosis. Moreover, genome-wide nucleosome positioning of ccfDNAs is utilized to infer pathological states of multiple disease types. A comprehensive public database called cell-free epigenome atlas (CFEA) provides the epigenome profile of ccfDNAs from various human diseases and can help in a better understanding of collected data (Yu et al., 2020). ccfDNA are generally associated with nucleosomes and histone proteins. Histone proteins are posttranslationally modified at amino acid residues located on their N- and C-terminal tails. These modifications act as epigenetic marks that can specifically distinguish disease-related ccfDNAs in blood samples. Various types of histone modifications are associated with the development and pathogenesis of human diseases (Zhao and Shilatifard, 2019).

In addition to DNA markers, RNA markers like mRNAs, miRNAs, lncRNAs, and circRNAs are also getting attention in the focus of clinical research (Pos et al., 2018).

Most of the currently available diagnostic tests based on ccfNAs use either DNA methylation markers or the differential expression of miRNAs. These biomarkers are relatively easily detected and estimated using accessible techniques like methyLight, methyl-specific PCR, methylation-sensitive high-resolution melting, and pyrosequencing (García-Giménez et al., 2017). DNA methylation specific to fetal and tumor DNA has been reported in pregnant women and cancer patients, respectively (Wong et al., 1999; Poon et al., 2002). The pattern of the methylation in these ccfDNAs has been traced back to their tissue of origin (Lun et al., 2013; Sun et al., 2017). Differentially methylated markers have been reported in ccfDNAs like INS promoter 1 in diabetes and *REG1A* and *CUX2* genes in pancreatic cancer (Lehmann-Werman et al., 2016). Promoter methylation of *SERPINB5*, *RASSF1A*, and *STAT5A* act as epigenetic fetal markers in maternal blood (Chim et al., 2005; Chan et al., 2006; Rahat et al., 2016a).

## Diagnostic Approach for Epigenetic Modifications in ccfNA

The various diagnostic approaches to study the epigenetic modifications in the nucleic acids include methylated CpG island recovery assay (MIRA) and MethylCap that rely on methyl-CpG-binding domains (MBD) to capture methylated DNA after DNA fractionation either by restriction digestion or sonication (Mitchell et al., 2011). These methods can also be combined with microarray or NGS technologies (MethylCap-seq) to identify biomarkers for cancer diagnosis and DNA methylation maps of cancer genomes (Simmer et al., 2012). Reduced representation bisulfite sequencing (RRBS) (Meissner et al., 2005) is an efficient method for absolute quantification of the methylation status of more than one million CpG sites at single base-pair resolution, covering regions of moderate to high CpG density (Lee et al., 2014). New techniques such as whole-genome bisulfite sequencing (WGBS) allows for an unbiased assessment of DNA methylation at single-base resolution with full coverage of more than 28 million CpG sites in the human genome, and by using this technique in the clinical settings, relevant biomarkers were identified in colorectal and breast cancers and certain types of leukemia (Berman et al., 2011).

Some of the techniques are used in clinical settings, like parallel shotgun sequencing and targeted sequencing (Norwitz and Levy, 2013) for non-invasive prenatal testing, WGS for fetal gene detection (Lo et al., 2010), and cancer personalized profiling by deep sequencing (CAPP-seq) to quantify circulating tumor DNA (Newman et al., 2014).

Despite the advancement of the techniques to study epigenetic modifications, the use of epigenetic biomarkers present on ccfNAs is limited due to their lower levels in the blood circulation. In the case of cancer, WGS is applied to only 5–10% of cell-free tumor DNA depending on the copy number. Mostly targeted methylation sequencing is carried out in such cases, which has a greater potential for the detection of lower levels of ccfNA in patients with early-stage disease.

Chromatin-based ChIP-seq experiments are revolutionizing our understanding of the complexes associated with chromatin dynamics. Ongoing advances such as nano-ChIP-seq allow ChIP-seq to be analyzed from far fewer cells necessary for embryology and development studies (Nakato and Shirahige, 2017). The emergence of ChIP-exo that digests the ends of DNA fragments not bound to protein is quite promising (Furey, 2012). However, the application of these techniques to identify biomarkers is limited due to the expertise and cost associated.

ChIP-seq also provides critical information on other chromatin modifiers, such as histone marks and the enzymes that modify these marks in diseases such as cancer. ChIP-seq has identified the role of aberrant H3K79 methylation by the methyltransferase DOT1L in mixed lineage leukemia (MLL)-rearranged leukemias (Bernt et al., 2011). In addition to ChiP-seq, different techniques like ChIP-PCR, ELISA-based assays, or mass spectrometry are used to detect and quantify histone modifications on ccfNAs in serum or plasma (Adli and Bernstein, 2011).

## ccfNAs as Epigenetic Biomarkers in Various Diseases

The detection and quantification of ccfNAs, viz. RNA, DNA, fetal DNA, fetal RNA, mtDNA, and mitochondrial RNA and miRNA levels in body fluids are of clinical significance. These ccfNAs have the potential to act as biomarkers for diagnosis as well as prognosis of various diseases (Fleischhacker and Schmidt, 2007; Breitbach et al., 2012), such as different cancers, obstetric, autoimmune, neurological, and mitochondrial diseases, as well as prenatal diagnosis, etc. (Kandel, 2012; Shaw et al., 2012). Although the most studied area of epigenetics is DNA methylation, yet in the clinical setting, there are only a few methylation markers. Various blood- or tissue-based cohort well-powered studies have recently shown that changes in the DNA methylation are not only observed frequently in cancers but also in other broad range of complex diseases including neurodegenerative, metabolic, autoimmune, and inflammatory diseases although at a lower frequency (Tost, 2016).

DNA methylation analysis of ccfDNA might provide a valuable option in some cases when the blood–brain barrier is temporarily disrupted. It was recently demonstrated by the detection of unmethylated fragments of *MBP3* and *WM1*, specific for oligodendrocytes in about 75% of patients with relapsing multiple sclerosis (Zachariah et al., 2009). cfRNAs are also present in the patient’s serum/plasma in addition to ccfDNAs. Higher levels of circulatory RNases were observed in cancers and various diseases like cerebral attack, preeclampsia, etc., and surprisingly, RNA found in the circulation was found to be stable (Umu et al., 2018). Changes in the expression of intracellular miRNA have been causally linked with many diseases that include cancer (Esquela-Kerscher and Slack, 2006), cardiovascular diseases (Navickas et al., 2016), neurodegenerative diseases (Gupta et al., 2017), etc. Such changes in expression of miRNA are either similar or distinct in the serum of a particular set of patients, thus enabling miRNA detection in serum as biomarkers of human diseases (Backes et al., 2016). Therefore, ccfNAs play a prominent role in the pathogenesis and diagnosis of various diseases. Further research is required in this field to ensure the widespread application of these markers in clinical settings.

## ccfNAs in Cancer

Every year, about 14 million new cases of cancer are reported (excluding skin cancer other than melanoma) that cause about 8.8 million deaths, accounting for 15.7% of deaths in a year (Ferlay et al., 2019). An estimated number of more than 1.8 million new cancer cases are likely to be diagnosed, and 606,520 cancer deaths are expected in the United States in 2020, which deciphers almost 1,660 deaths per day (Siegel et al., 2020). The six major hallmarks of cancer (Hanahan and Weinberg, 2000) are uncontrolled cell growth and division, programmed cell death avoidance, invasion, metastasis, and angiogenesis. The diagnosis of cancer usually occurs following the appearance of signs or symptoms or through screening and investigations like X-rays, blood tests, endoscopy, CT scans, etc. Biopsy tissue examination indicates the type of proliferating cells, genetic abnormalities, and histological grade, and other characteristics. Therefore, advanced measures such as estimating prognosis, risk assessment for early diagnosis, biomarkers, and observing the response to therapy can lead to successful treatment, positive outcomes, and improvement of the quality of life for patients. The tissue biopsy-matched ccfDNA is considered as surrogate marker due to its release from the tumor sites (De Mattos-Arruda et al., 2013). It is proven to be a non-invasive, rapid, and sensitive marker for diagnosis, prognosis, and therapy response monitoring in different cancers (Volik et al., 2016). In addition, the integrity of ccfDNA (extent of ccfDNA fragmentation) may be utilized as a promising biomarker for diagnosis and prognosis of cancer (Madhavan et al., 2014).

### ccfNAs as Diagnostic and Prognostic Biomarkers for Cancer

Serum or plasma ccfNA serves as a “liquid biopsy,” which is useful for various applications in diagnostics and avoids the necessity for biopsy of tumor tissue. The levels of ccfNA in blood and lymphatic circulation are affected by degradation, clearance, and various other physiological events. Liver and kidney clear nucleic acids from the blood, and they have a half-life of different time intervals in the circulation that varies from 15 min to several hours (Fleischhacker and Schmidt, 2007). miRNAs appear to be extremely stable, but their rate of clearance from the blood is not well studied in cancer patients thus owing to the uniqueness of this research area.

#### ccfDNAs in Cancer

ccfDNAs consists of both genomic DNA (gDNA) as well as mtDNA. There is a production of longer uneven fragments of DNA by necrosis in cancer patients and shorter DNA fragments from apoptosis. Hence, increased levels of longer DNA fragments in the bloodstream have been targeted as a potential marker for the presence of malignant tumor DNA (Arko-Boham et al., 2019). Tumor cells are the origin of ccfDNA in the blood of cancer patients (Stroun et al., 1989). Aberrations specific to tumors like oncogene and tumor suppressor gene mutations (Wang et al., 2004), methylation of DNA (Fujiwara et al., 2005), and instability of microsatellite DNA (Shaw et al., 2000) were recognized in ccfDNA. Tumorigenesis and its progression are monitored by the change in various epigenetic modifications. Patients with different types of malignancies have methylated DNA in their serum or plasma. One of the most important methods for analyzing malignancy is by detecting the presence of methylated ccfDNA in cancer patients.

For early diagnosis of colorectal cancer (CRC), analysis of promoter hypermethylation in blood and fecal DNA has the potential to be used as a non-invasive test, and efforts are made for clinical application of these molecular markers. Various studies have observed *MGMT*, *RASSF2A*, *Wif-1*, *NGFR*, and *SEPT9* as aberrantly methylated genes used as diagnostic biomarkers in patients with CRC (Lee et al., 2009; Powrozek et al., 2014). Several potential methylation biomarkers have been found that differentiate plasma from breast cancer patients and that from control subjects (Hoque et al., 2006). Remarkably, two independent studies recognized *CST6* as being methylated differentially between breast cancer and control plasma samples (Radpour et al., 2011; Chimonidou et al., 2013). For lung cancer, an early focus was to search methylated *CDKN2A* as a plasma diagnostic biomarker. Studies observed hypermethylation of *CDKN2A* in the plasma of patients with lung cancer as compared to cancer-free controls (Zhang et al., 2011). *SHOX2* was identified as a potential biomarker in a retrospective study done by researchers from the Theracode, a diagnostic firm (Kneip et al., 2011). A recent study, by a group, as part of the Australian Pancreatic Cancer Genome Initiative (APGI), has observed elevated levels of aberrant methylation in the important cell signaling pathways, thus suggesting its possibility as a disease biomarker. They worked on a group of six candidate genes, *NPTX2*, *SARP2*, *UCHL1*, *ppENK*, *CDKN2A*, and *RASSF1A*, and observed differential methylation in the promoters of all the genes in pancreatic cancer and healthy controls except in *CDKN2A* promoter, which was methylated differentially between pancreatic cancer patients and those having chronic pancreatitis (Park et al., 2012). Epigenetic events in the progression of cancer include the promoter region hypermethylation of the genes, pi-class *GSTP1*, and *APC*, which are the most common somatic genome abnormalities in colorectal and prostate cancer (Ellinger et al., 2008). *RASSF1A*, *RARB*, *SEPT9*, *ESR1*, and *CDKN2A* are the important methylated genes that have shown utility in prognosis using ccfDNA assays in many patients. Methylation of histones is an active process with vital roles in differentiation and development. Tumorigenesis also occurs due to aberrant levels of histone methylation. The promoters associated with H3K4 are primarily trimethylated by *SET1A* and *SET1B*. *SET1A* plays a vital role in oncogenic function in breast cancer metastasis, lung cancer, and colorectal cancer (Zhao and Shilatifard, 2019). Table 1 presents the frequently hypermethylated genes in various cancer types.

**TABLE 1:** Frequently hypermethylated genes in various cancer types.

**Gene**	**Cancer Type**	**References**
*ITIH5, DKK3, BRCA1, ER-beta, APC, GSTP1, ESR-b*	Breast cancer	Kloten et al., 2013; Cheuk et al., 2017; Vu et al., 2018
*RASSF1A*	Prostate cancer	Liu et al., 2002
*P16*	Esophageal, liver, and pancreas	House et al., 2003
*ARF, BAX, BCL2, CDH1, DAPK, EDNRB, EOMES, FADD, PCDH17, POU4F2*	Bladder cancer	Abern et al., 2014; Wang et al., 2016
*SEPT9, HLTF, NELL1, CEA, TAC1*	Colorectal cancer	Tham et al., 2014; Semaan et al., 2016
*VHL*	Kidney tumors	Ma et al., 2017
*RB*	Retinoblastoma	Ohtani-Fujita et al., 1997
*TMEFF2, PRDM1,3OST2, MGMT*	Lung cancer	Palmisano et al., 2000; Lee et al., 2012; Su et al., 2016
*APC, GSTP1*	Renal cell carcinoma	Hauser et al., 2013
*ST6GALNAC3, ZNF660*	Prostate	Haldrup et al., 2018
*BRCA1, RASSF1A, RASSF2A*	Ovarian cancer	Giannopoulou et al., 2017; Lonning et al., 2018
*hTERT*	Leptomeningeal carcinomatosis in CSF	Bougel et al., 2013
*p16INK4a, TIMP-3, THBS1*	Glioma	Liu et al., 2010

#### CcfmiRNAs in Cancer

In various cancers, miRNA expression dysregulation has been observed that suggests its role in many processes necessary for the progression of cancer like proliferation, cell death, metastasis, and resistance to treatment (Iorio and Croce, 2012). During the development of the liver, miRNA expression changes dynamically. *miR-500* is one such oncofetal miRNA that is important for the diagnosis of hepatocellular carcinoma (Yamamoto et al., 2009). Lately, in non-small cell lung cancer (NSCLC), *miR-1246* and *miR-1290* were recognized as tumor-initiating and cell-specific miRNAs (Zhang et al., 2016). *miR-1290* was found to be a significant prognostic factor for OSCC patients based on Cox regression analysis. In addition, *miR-1290* could serve as a valuable biomarker in OSCC patients to predict the clinical response to chemoradiotherapy (Lin et al., 2018). A study by Alhasan et al., 2016 showed a serum signature of 5-miRNAs (*miR-135a*, *miR-106a*, *miR-200c*, *miR-605*, and *miR-433*) predicted a very high-risk prostate cancer (Alhasan et al., 2016). Expression levels of *miR-21*, *miR-23b*, *miR-200c*, and *miR-200b* were upregulated in metastatic breast cancer when compared to early breast cancer patients, therefore supporting the notion that ccfmiRNAs presents a tool with the crucial diagnostic and prognostic implication in breast cancer (Papadaki et al., 2019). Furthermore, a study discovered that increased *miR-122* expression was significantly associated with a reduction in the overall survival as well as progression-free survival in breast cancer patients (Saleh et al., 2019). Elevation in the levels of serum *miR-29*, *miR-122*, *miR-155*, and *miR-192* was observed in cholangiocarcinoma. Although miRNAs levels before surgery were inappropriate as survival prognostic marker; however, postsurgery decrease in the serum *miR-122* levels was significantly linked with better patient prognosis (Loosen et al., 2019).

### ccfNAs in Treatment and Cancer Progression

ccfDNA analysis is a non-invasive process that allows day to day patient follow-up and monitoring of response toward treatment (Gorges et al., 2012). Both genetic and epigenetic changes are exhibited by ccfDNA (Stroun et al., 2001). The study of these changes might provide valuable information to mold the choice of treatment by clinicians given the limitations of the novel targeted therapies.

Abnormal hypermethylation at CpG islands occurs rarely in non-malignant and normally differentiated cells, so the release of DNA from tumor cells can be found with a prominent extent of sensitivity, even when the excess of DNA is released from normal cells, and this characterizes its potential clinical application (Wong et al., 2001). In this context, promoter region hypermethylation of *INK4A* occurs very early in the progression of hepatocellular carcinoma (HCC), and hence, it serves as a valuable biomarker for non-invasive diagnosis as well as prediction of response to therapy (Huang et al., 2014).

In the *MYCN*-amplified neuroblastoma progression, *MYCN* is detected in circulating DNA. This phenomenon was found to be associated strongly with the quick progression of tumors and poor outcomes (Combaret et al., 2002). Loss of heterozygosity (LOH) and abnormal methylation at the promoter region of *MYCN* were detected using ccfDNA, which showed elevated levels in patients of high-grade glioma. Detection of promoter region hypermethylation of *MYOD1* in serum may serve as a potential prognostic marker for discriminating patients of cervical cancer at high risk for lymph node metastasis or relapse (Widschwendter et al., 2004).

Moreover, the investigation of circulating miRNAs presents great potential in revealing new insights into their role in therapy and diagnosis. miRNA serum signatures (*miR-345 -5p*, *miR-330 -3p*, and *miR-9 -3p*) were found to be significantly upregulated in patients of prostate cancer (PCa) when compared to healthy individuals. The role of *miR-345-5p* to act as an oncomir through *CDKN1A* targeting makes it a potential target for PCa therapeutically (Tinay et al., 2018).

Immunotherapy is a rapidly developing therapy in many cancers because of various advantages over standard chemotherapy. Identification of significant miRNAs that provides a foresight of response in cancer immunotherapy would enable better patient selection and enhancement of therapeutic efficacy and provide a novel target (Antonia et al., 2004; Chen et al., 2008). *miRNA-21* is a cell-free oncogenic miRNA, which has been known as a potential regulator of *STAT3*, and thus, it could be detected in various tumors (Ji et al., 2009). Thus, circulating *miRNA-21* can act as a biomarker for response in cancer immunotherapy (Wu et al., 2012).

ccfDNAs in glioma were associated with differential methylation levels of *MGMT*, cyclin-dependent kinase inhibitor 2A, multiple tumor suppressor 1 *p16/*(*INK4a*), *p73*, and retinoic acid receptor beta (*RARb*) (Balana et al., 2003; Weaver et al., 2006; Wakabayashi et al., 2009). All these studies propose a crucial role of epigenetic marks in ccfNAs in cancer-targeted therapy as well as pathogenesis.

### ccfNAs in Cancer Precision Medicine

Precision oncology is an approach that includes the molecular profiling of tumors to identify effective therapeutic strategies. A clinical research program initiated by The Englander Institute for Precision Medicine (EIPM) in 2013 uses whole-exome sequencing of metastatic and primary tumors to identify individualized therapeutic options and to help guide clinical decision making, by prospective follow-up of patients (Rennert et al., 2016). Oncology is the obvious choice for heightening the impact of precision medicine. Several targeted therapies have been developed that have shown profound benefits. Recently, novel immunological approaches produced insightful responses (Snyder et al., 2014).

In addition, the identification of epigenetic biomarkers leads to more precise disease prognosis, especially in therapeutic areas that are linked with a high degree of variability concerning survival (Van Neste et al., 2017). Research carried out in several cancers like glioblastoma reveals that levels of 5hmC are critical in the regulation of genes having a crucial role in disease and show that global reduction in 5hmC over the genome leads to poor clinical outcomes in these patients (Johnson et al., 2016).

Epigenetic changes introduced common genetic mutations in an *in vitro* model of lung cancer (Vaz et al., 2017). Epigenetic-based diagnostics can detect early disease signals and thus can provide possibilities for clinical intervention before the progression of symptoms.

The detection of ccfNAs could be exploited by targeted therapies approved lately and eventually benefit the patients. Scrutinizing cancers by analyzing ccfNA dynamics in blood or serum is an innovative and emerging research area. As far as the existing research advancement and the growth of the medical industry are concerned, we consider that ccfNA assays may be employed for real-time personalized treatments in the future for cancer patients, based on their ccfNAs or ccfDNA methylation levels, for diagnosis and prognosis. Nevertheless, there is much scope for improvement before the application of this technology in clinical settings.

## Use of ccf-Fetal-NAs in Prenatal Diagnosis and Pregnancy-Related Disorders

During pregnancy, the apoptosis/necrosis of trophoblasts arising from syncytiotrophoblast is the prime source of the release of ccf-fetal-NAs into the maternal blood (Litton et al., 2009). The presence of ccf-fetal-NAs has paved the way for non-invasive prenatal diagnosis and early prediction of pregnancy-related complications (Lo et al., 1997, 1998). The use of ccf-fetal-NAs has gradually replaced invasive techniques like amniocentesis or chorionic villus sampling (Serr et al., 2017). ccf-fetal-DNA comprises 10–15% of the maternal ccfDNA (Wang et al., 2013) and can be efficiently detected at the fifth week of gestation (Guibert et al., 2003). The amount of ccf-fetal-DNA in maternal blood increases progressively throughout pregnancy (Birch et al., 2005).

### ccfNAs in Prenatal Diagnosis

Prenatal diagnosis is an established practice for the management of pregnancy as well as avoidance of prenatal/neonatal deaths. The leading causes for such deaths are genetic disorder, birth defects, congenital malformations, and chromosomal abnormalities like trisomy 21 (Down’s syndrome), 18 (Edward’s syndrome), and 13 (Patau syndrome), and sex chromosome aneuploidies like monosomy X (Turner syndrome) (Carlson and Vora, 2017). Therefore, successful management of pregnancy demands efficient and timely prenatal diagnosis to determine the outcome of pregnancy. Timely detection of neural tube defects is already providing early prenatal treatment resulting in better neonatal outcomes (Adzick et al., 2011).

ccf-fetal-DNA is clinically used for the detection of fetal sex and multiple anomalies based on paternally inherited mutations (Bianchi, 1998). Recent studies have discovered many fetal epigenetic biomarkers for ccf-fetal-NA-based liquid biopsies in clinical samples that have demonstrated high clinical potential in disease diagnosis, prognosis, and pregnancy management. These epigenetic modifications are specific to the fetus and help to distinguish fetal nucleic acids from maternal nucleic acids (Jones and Takai, 2001). Clinical testing of recently developed fetal epigenetic markers can help in the proper management of personalized care. The first reported use of fetal-derived epigenetic marker in maternal body fluids had come from Poon et al. (2002) who utilized an imprinted *H19/Igf2* locus based on parent-of-origin-specific methylation, and the maternal and the paternal copies of the gene were distinguished in maternal blood (Poon et al., 2002). Based on the placental origin of ccf-fetal-DNA having placenta-specific DNA methylation pattern, the genomic regions that show differential methylation between the placenta and the maternal blood cells can act as a marker for fetal DNA in maternal blood. The promoter region of *maspin* (*SERPINB5*) is the first such reported universal fetal DNA marker, with detectable hypomethylation, in the background of hypermethylated maternal sequences. The fetal origin of these hypomethylated *maspin* has been confirmed by the clearance of these sequences within 24 h of delivery (Chim et al., 2005). The clinical use of hypomethylated *maspin* is limited by the required bisulfite treatment of ccf-fetal-DNA, as this treatment can degrade around 95% of the DNA (Grunau et al., 2001), thus decreasing the amount of already low levels of fetal DNA in maternal blood. Such limitation was overcome by the detection of fetal-derived hypermethylated *RASSF1A* in maternal blood for prenatal diagnosis (Chan et al., 2006; Hyland et al., 2009; Tounta et al., 2011b). The maternal hypomethylated *RASSF1A* ccfDNA can be removed by treatment with methylation-sensitive restriction enzyme digestion, leaving behind fetal hypermethylated *RASSF1A* ccf-fetal-DNA (Chan et al., 2006). Various other fetal-derived differentially methylated sequences have also shown a similar potential to act as fetal DNA epigenetic markers in maternal blood (Table 2).

**TABLE 2:** Clinical application of ccf-fetal nucleic acids in prenatal diagnosis and pregnancy-related diseases.

**Clinical application**	**Gene**	**Detection method**	**References**
Fetal DNA marker	SERPINB5	Hypomethylated ccf-fetal DNA	Chim et al., 2005
	RASSF1A; APC and PRKCDBP; MEST and SNRPN	Hypermethylated ccf-fetal DNA	Chan et al., 2006; Rahat et al., 2016b; Rahat et al., 2017a
Fetal Rh status	RASSF1A	Hypermethylated ccf-fetal DNA	Hyland et al., 2009; Tounta et al., 2011b
Trisomy 21	HLCS	Fetal DNA allelic ratio	Tong et al., 2010
Trisomy 18	SERPINB5		Tong et al., 2006
Congenital heart diseases	miR-19b, miR-22, miR-29c, and miR-375, miR-99a	↑ level of fetal miRNAs	Zhu et al., 2013; Kehler et al., 2015
	ENST00000436681, ENST00000422826, AA584040, AA706223, and BX478947	De-regulated lncRNAs	Gu et al., 2016
Preeclampsia	SERPINB5	↑ level of hypomethylated ccf-fetal DNA.	Chim et al., 2005
	RASSF1A	↑ level of hypermethylated ccf-fetal DNA	Tsui et al., 2007
	c-myc	Hypermethylated ccf-fetal DNA	Rahat et al., 2014
	VEGF	Hypermethylated ccf-fetal DNA	Rahat et al., 2017b
	Corticotrophin-releasing hormone	↑ level of fetal mRNA	Hahn et al., 2011
	miR-1233, miR-520, miR-210, miR-155	↑ level of fetal miRNAs	Ura et al., 2014; Nagy, 2019
	miR-144	↓ level of fetal miRNA	Ura et al., 2014
	miR-24, miR-26a, miR-103, miR-130b, miR-181a, miR-342-3p, and miR-574-5p	↑ level of fetal miRNAs	Wu et al., 2012; Barchitta et al., 2017
	miR-26a and miR-342-3p	↑ level of fetal miRNAs	Choi et al., 2011
IUGR	miR-518b and miR-519a	↑ level of fetal miRNAs	Wang et al., 2014
	–	↑ level of ccf-fetal DNA	Romao et al., 1992; Sifakis et al., 2015
Preterm birth	miR-143 and miR-145	↑ level of fetal miRNAs	Elovitz et al., 2014
	miR-200a, miR-4695-5P, miR-665, and miR88	Altered structure of fetal miRNAs	Elovitz et al., 2015
Hyperemesis gravidarum	–	↑ level of ccf-fetal DNA	Romao et al., 1992
Placenta accrete/inccreta	–	↑ levels of ccf-fetal DNA	Romao et al., 1992
Gestational diabetes mellitus	miR-518d, miR-508-3p, miR-27a, miR-9, miR-137, miR-92a, miR-33a, miR-30d, miR-362-5p, and miR-502-5p	↑↓ level of fetal miRNAs	Grissa et al., 2010; Barchitta et al., 2017
Low birth weight infants	mir-517a	↑ level of fetal miRNAs	Song et al., 2013
	–	↑ level of ccf-fetal DNA	Sifakis et al., 2015

**IUGR, intrauterine growth restriction*.*

ccf-fetal-DNA methylation markers have the potential of being used as both quantitative as well as qualitative markers in prenatal diagnosis. As qualitative markers, these are used to estimate the false positives during the determination of fetal gender, Rh status, and paternally inherited polymorphisms (Chan et al., 2006), while as quantitative markers, these can estimate the levels of ccf-fetal-DNA in maternal plasma. Such an application of ccf-fetal-DNA finds its use in the detection of chromosomal aneuploidies (Lun et al., 2008). Based on the location of the *maspin* gene on chromosome 18, hypomethylated fetal *maspin* has been used to calculate the allelic ratio to diagnose trisomy 18 with 100% sensitivity (Tong et al., 2006). Fetal trisomy 21 was detected by analyzing chromosomal dosage via targeting of fetal hypermethylated *HLCS* sequences in the combination of microfluidics digital PCR. *RASSF1A* on chromosome 3 and *ZFY* on the Y chromosome were used as references (Tong et al., 2010). Fragmentation pattern of ccf-fetal-DNA in maternal plasma has been successfully used for enrichment method in size separation manner on agarose gel electrophoresis (Ramezanzadeh et al., 2017).

Various next-generation sequencing and high-throughput techniques have catalyzed the identification of newer and novel fetal epigenetic markers further advancing non-invasive prenatal diagnosis. The microarray-based approach has identified many fetal epigenetic markers with differential methylation between chorionic villus samples and maternal blood, on chromosomes 21, 13, and 18 for aneuploidy detection (Chu et al., 2009). Combining high-resolution tiling oligonucleotide array with methylated DNA immunoprecipitation (MeDiP) has helped in a genome-wide screen for detecting the differential methylated sites between placental tissue and maternal blood cells. It has detected various new fetal epigenetic markers on chromosomes 21, 13, and 18 (Papageorgiou et al., 2009). Whole-genome bisulfite sequencing has further identified many clinically useful novel fetal-specific methylated CpG sites (Lun et al., 2013). Latest techniques like high-resolution methylation-specific bead chip microarray (Hatt et al., 2015) and GeneChip Human Promoter 1.0R Array (Wang et al., 2017) identified many differentially methylated CpG sites between maternal blood cells and chorionic villi, which can help in better and efficient prenatal diagnosis and the expansion of its application in other disorders.

More recently, non-coding RNAs like miRNAs, lncRNAs, and circRNAs are in the focus of the clinical research for prenatal diagnosis. Several placenta-specific miRNAs are differentially expressed within the placenta and are also secreted during pregnancy from the trophoblast layer of the placenta (Table 2). These are located in clusters on chromosomes 14 and 19 (*C14MC*, *C19MC*, and *miR-371-3*) (Nagy, 2019). Highly stable placental miRNAs were detected in maternal plasma. These can help in tracking gene regulation in the placenta (Chim et al., 2008). ccf-fetal miRNAs in the maternal blood act as expression-based novel epigenetic markers in prenatal diagnosis. miRNA microarray-based screen has detected many differentially expressed ccf-fetal miRNAs in maternal serum to diagnose congenital heart defects, which can be especially helpful in personalized care (Gu et al., 2019). Circular RNA and lncRNA are used in prenatal diagnosis for congenital heart diseases (Nagy, 2019). Early diagnosis of congenital heart diseases is beneficial to reduce morbidity and mortality. Sequencing by Oligonucleotide Ligation and Detection (SOLiD) has been used to identify congenital heart disease-related miRNAs in maternal blood (Zhu et al., 2013; Kehler et al., 2015). Gu et al. (2016) has reported many lncRNAs related to congenital heart diseases. The clinical use of these lncRNAs is extensively studied (Gu et al., 2016).

The use of artificial intelligence platforms and machine learning in combination to analyze genome-wide DNA methylation data has helped to identify epigenomic predictors for cerebral palsy in newborns with better sensitivity and specificity. These epigenetic predictors provided more mechanistic information about the pathogenesis of cerebral palsy (Bahado-Singh et al., 2019).

### ccfNAs in Pregnancy-Related Disorders

Certain pregnancy-related complications are associated with poor placental growth and development, which is usually also accompanied by aggravated trophoblastic apoptosis, resulting in the release of increased amounts of ccf-fetal-NAs into maternal blood. ccf-fetal-NAs are especially important for precision medicine being useful for clinical diagnosis and management of these complications, as these precede the actual clinical symptoms of the disease (Sifakis et al., 2015). The quantification of ccf-fetal-DNA levels in maternal blood might serve as an indicator of some developing abnormality; however, the absolute concentration of ccf-fetal-DNA varies with maternal weight and ethnicity and fluctuates throughout pregnancy (Tounta et al., 2011a), which warrants the need for a disease-specific qualitative marker. Aberrations in the levels of epigenetic marks present in these fetal DNA fragments serve as a valuable alternative for the diagnosis and management of pregnancy-related complications. The ccf-fetal-DNA present in maternal blood has been explored to predict such placental abnormality-linked pregnancies, like intrauterine growth restriction (IUGR); preeclampsia; hemolysis, elevated liver enzyme levels, and low platelet levels (HELLP) syndrome; preterm labor; hyperemesis gravidarum (severe morning sickness); placenta accrete; and placenta inccreta (Romao et al., 1992).

IUGR, defined by less than fifth percentile fetal weight, may or may not be associated with preeclampsia. Early detection of preeclampsia is highly beneficial for the proper management of preeclampsia, which is highly important for both the developing fetus and the mother (Bauer et al., 2006). The development of preeclampsia is also depicted by the increased level of unmethylated fetal *SERPINB5* (Chim et al., 2005) in maternal blood. Similarly, hypermethylated fetal *RASSF1A* (Tsui et al., 2007) sequences are also elevated in preeclamptic blood. Hypermethylated *c-myc* and *VEGF* observed specifically in ccf-fetal-DNA in preeclampsia patients are the epigenetic markers, which can diagnose preeclampsia without the requirement of quantitative estimation of ccf-fetal-DNA levels. These sequences can be used both for the diagnosis as well as prognosis of preeclampsia (Rahat et al., 2014, 2017b). Such ccf-fetal-DNA-based epigenetic markers can be beneficial for early prediction and the personalized management of the disease. Fetal DNA epigenetic markers are likely to show potential as diagnostic markers in other complicated pregnancies accompanied by quantitative aberrations of ccf-fetal-DNA (Tsui et al., 2010).

Several fetal-derived mRNAs and miRNAs also as serve as diagnostic and prognostic markers for preeclampsia, preterm births, IUGR, spontaneous abortions, and low birth weight infants. A list of clinical applications of ccf-fetal nucleic acids in prenatal diagnosis and pregnancy-related diseases are given in Table 2. In addition, miRNAs involved in impaired trophoblast migration and invasion (*miR-195*, *miR-276C*, *miR-278a-5p*, and *miR-210*), impaired angiogenesis (*miR-210*, *miR-21*, and *miR-22*), and dysregulation of the maternal immune system are associated with preeclampsia (Skalis et al., 2019). Aberrant expression of circular miRNAs reported in gestational diabetes mellitus can serve as potential biomarkers for early diagnosis (Barchitta et al., 2017). Microarray analysis has also identified many miRNAs related to gestational diabetes mellitus (Grissa et al., 2010).

The use of microarray and next-generation sequencing can help to identify more ccf-fetal-RNA markers (Ferrari et al., 2008). Extensive research is required on different non-coding RNAs to be utilized in clinical settings for early diagnosis of pregnancy-related disorders.

The major obstacles in the field of ccf-fetal-NAs for diagnosis of prenatal and pregnancy-related complications are the requirements for proper standardized protocols for sample processing, detection methods, data analysis, and appropriate quality controls. Low concentration and fragmented pattern of ccf-fetal-NAs further demand the development of novel technologies for the proper utilization of ccf-fetal-NAs for diagnostics. The combined use of next-generation sequencing and bio-informative analysis could facilitate large-scale comprehensible screening and identification of promising next-generation non-invasive epigenetic biomarker in ccf-fetal-NAs. The screening for novel and disease-specific epigenetic markers on ccf-fetal-NAs in maternal blood will not only help in early diagnosis but also in providing proper personalized care. The ccf-fetal-NA-based diagnostic techniques provide new highly sensitive and specific avenues in clinical settings. These have already replaced invasive diagnostic sampling reducing pregnancy risks. ccf-fetal DNA is already being used in clinical settings, while the use of lncRNAs, circular RNAs, and miRNAs are in the research phase and could soon be used for clinical diagnosis of many fetal- and pregnancy-related disorders.

Intensive research is required in this area based on large populations to develop new clinical applications of fetal epigenetic marks in maternal blood. Additional mechanistic studies are required to identify the epigenetic changes behind the fetal–maternal complications, which can provide more insight into the possible epigenetic marks for non-invasive diagnosis.

## ccfNAs in Autoimmune Diseases

In autoimmune diseases, there are abnormal immune responses to healthy body tissues. In the United States, about 8% of the population (24 million) are affected by autoimmune diseases, where women are more commonly affected as compared to men (Fairweather and Rose, 2004). Nearly 80 different types of autoimmune diseases are known (Hayter and Cook, 2012). The appearance of disease symptoms in adulthood makes the diagnosis of autoimmune immune diseases difficult. Celiac disease, Graves’ disease rheumatoid arthritis, systemic lupus erythematosus, diabetes mellitus type 1, inflammatory bowel disease, and multiple sclerosis are some well-known autoimmune diseases (Hohlfeld et al., 2016).

### ccfNAs as Epigenetic Markers in Diagnosis, Progression, and Treatment of Autoimmune Diseases

Witebsky’s et al. (1957) postulates were formulated for the first time for the diagnosis of autoimmune diseases. Accumulating evidence has shown that there is a significant role of epigenetic modifications in the development and progression of autoimmune diseases. With the benefits of ease of detection and the ability to analyze disease activity, specific epigenetic modifications can be proposed as novel biomarkers in autoimmune diseases.

In autoimmune diseases like other several pathological conditions, the presence of ccfDNA, has been observed, thus developing the interest of using them as a potential biomarker. Many pieces of evidence have shown that there is the presence of abnormal DNA demethylation in peripheral blood mononuclear cells (PBMCs) and CD4+ T cells of lupus patients (Javierre et al., 2010; Jeffries et al., 2011; Hewagama et al., 2013). Hypomethylation status of two sites, CpG site1 (Chr1: 79,085,222) and CpG site 2 (Chr1: 79,085,250; cg06872964), within the promoter region of IFI44L (IFN-induced protein 44-like) were identified as biomarkers for the diagnosis of SLE and further validated in the Chinese population consisting of 1,144 lupus patients, 1,350 healthy subjects, 429 RA, patients and 199 patients of primary Sjögren’s syndrome (pSS), as well as in a European cohort (Zhao et al., 2016). DNA methylation levels thus can not only distinguish active patients from inactive ones but importantly also indicate the activity of autoimmune diseases.

DNA methylation might be a good parameter, different from genetic and protein biomarkers, to serve as a predictive biomarker. Reduced DNA methylation at the *IL-6* and *ERa* promoters in PBMCs in RA patients is associated with overexpressed *IL-6* and hyperactive *ERa* signaling (Nile et al., 2008; Liao et al., 2012; Liu et al., 2014). Aberrant epigenetic modifications also have been evidenced to be associated with systemic sclerosis (SSc) disease. In this context, abnormal global and gene-specific DNA demethylation (e.g., at *CD11a*, *CD70*, and *CD40L*) and several hypermethylated genes (*PRF1*, *CD11a*, *FoxP3*, *CD70*, and *CDKN2A*) in whole blood have been observed from South Africans with SSc (Matatiele et al., 2015). Besides, the Th17-related genes, like *RORC1* and *RORC2*, are hypomethylated from SSc patients in PBMCs and further correlated with inflammatory parameters (Almanzar et al., 2016). However, these aforementioned alterations are not validated as predicted biomarkers as yet, so extensive work is required in this direction in the future. Overall, these studies suggest that changes in circulating DNA methylation levels, observed in autoimmune diseases (Rainer et al., 2003), can act as an important tool to monitor the response of the treatment and predict progression of the disease and patient’s stratification according to different stages in the disease.

Like cell-free DNA, a few studies have also reported the role of cell-free RNAs in autoimmune diseases. Recent studies have revealed that there are ∼600 circulatory RNAs differentially expressed in the PBMCs in patients suffering from rheumatoid arthritis (Zheng et al., 2017). Similarly, over 200 circulatory RNAs in the plasma of SLE patients and 400 in the PBMCs of relapsing–remitting multiple sclerosis patients (RR-MS) were found in comparison to healthy controls (Cardamone et al., 2017). Such an abundance of different circulatory RNAs can help improve the efficiency of clinical diagnosis by their combined detection with other transitional markers.

Apart from these two types of ccfNAs, other forms like miRNA, long non-coding RNA, and mtDNA also have tremendous potential in future screening, diagnosis, and prognosis of autoimmune diseases. Many studies have identified miRNAs that are abnormally expressed in lupus. However, target genes have been identified only for a few of them. In a recent study, circulating miRNA profile was identified in patients with autoimmune disease as well as in Treg-depleted mice model. Results of this study from quantitative reverse transcription PCR (qRT-PCR) quantification and analysis of receiver operating characteristic (ROC) curve were able to determine a total of six miRNAs (*miR-551b*, *miR-34cmiR-448*, *miR-9*, *miR-148*, and *miR-124*) in the mouse models with T-reg depletion and three miRNA (*miR-448*, *miR-124*, and *miR-551b*) in patients with RA, SLE, Sjogren’s syndrome (SS), and ulcerative colitis (UC), leading to a conclusion that they could serve as valuable specific biomarkers in these diseases.

As the optimal source of biomarkers, many other circulating miRNAs also have been identified to be correlated with lupus. Among them, *miR-146a* and *miR-155* are the first miRNAs that have been described as decreased in lupus serum (Wang et al., 2010). In subsequent studies, the serum levels of *miR-200a*, *miR-200b*, *miR-200c*, *miR-429*, *miR-205*, *miR-192*, *miR-126*, *miR-16*, *miR-451*, *miR-223*, *miR-21*, and *miR-125a-3p* (Wang et al., 2011, 2012) were found to be abnormally expressed in SLE and correlated with disease activity.

Another inspiring observation is that *miR-126* has been reported to regulate DNA methylation in lupus T cells by targeting DNMT1 (Zhao et al., 2011), supporting the idea that lupus T cells are switched on by DNA hypomethylation via miRNAs (Ceribelli et al., 2011). Future studies in this direction can establish that not only circulatory DNA or RNA but also circulating miRNAs can also represent potential universal epigenetic biomarkers for autoimmune diseases (Jin et al., 2013).

At present, the treatment for autoimmune diseases is primarily based on immunosuppressive as well as anti-inflammatory agents, which mostly include humanized monoclonal antibodies, engineered biologics, and fusion proteins. Such treatment options are particularly generated against some signaling pathways or are selective for a certain subset of cells in the immune systems. The effectiveness of such types of treatments is for short duration only and not even antigen-specific in some cases. Moreover, chronic administration of these agents leads to the common side effects of increased incidence of infections and general immunosuppression (Tavakolpour, 2017). By utilizing the disease-specific epigenetic marks on the ccfNAs, for non-invasive detection, monitoring, and screening of autoimmune disorders, it will become feasible to offer personalized medicine to manage the autoimmune diseases in the future. A list of ccfNAs in autoimmune diseases is presented in Table 3.

**TABLE 3:** Cell-free nucleic acids in autoimmune diseases.

**Autoimmune disease**	**Cell-free/circular nucleic acids**	**Source for diagnosis**	**No. of patients involved in study**	**References**
SLE	cfDNA	Serum	95	Tan and Kunkel, 1966
RA and SLE	cfDNA	Synovial fluid and serum	14	Barnett, 1968
RA and SLE	cfDNA	Derum	114	Koffler et al., 1973
RA	cfDNA	Serum	70	Leon et al., 1977
SLE	cfDNA	Serum and plasma	12	Chen et al., 2007
SLE, RA, and SS	cfDNA	Plasma	112	Bartoloni et al., 2011
RA	CircRNA	PBMCs from blood	20	Zheng et al., 2017
SLE	CircRNA	Plasma	30	Li et al., 2018
RR-MS	CircRNA and DNA	Blood and tissues	30 (DNA) 10 (RNA)	Cardamone et al., 2017
RA, SLE, SS, and UC	Circulating miRNA	Serum	103	Jin et al., 2018

**RA, rheumatoid arthritis; RRMS, relapsing–remitting multiple sclerosis; SLE, systemic lupus erythematosus; SS, Sjogren’s syndrome; LN, lupus nephritis; UC, ulcerative colitis; circ, circular*.*

## ccfNAs in Neurological Diseases

Neurological disorders include diseases associated with central as well as the peripheral nervous system. These disorders include Alzheimer’s disease (AD), epilepsy, other dementias, Parkinson’s disease (PD), and traumatic disorders of the nervous system (Amor et al., 2014).

Symptoms associated with chronic neurodegenerative diseases occur late after the beginning of the pathology due to the compensatory potential of the brain that has been demonstrated in various studies. Any treatment is difficult in the later stages of neurodegenerative diseases due to the massive death of neuronal cells (Sperling et al., 2011; Pillai and Cummings, 2013). The recent ability to detect neurological biomarkers in the blood is due to technological advances in detection. The advances related to the use of ccfNA in neurological disorders are as follows.

### ccfNAs in Diagnosis, Progression, and Treatment of Neurological Diseases

Cell-free DNA acts as a marker for traumatic brain injury (TBI) and neurodegenerative diseases. The blood–brain barrier gets disrupted and leaky after TBI and neurodegenerative diseases (Carvey et al., 2009), which makes ccfNAs as potential markers for disease as well as injury (Lehmann-Werman et al., 2016). This includes changes in levels of ccfNA overall and also ccfNA markers specifically associated with the brain (Zlokovic, 2011).

Specific cell-free miRNA levels (*mir-34c*) involved in apoptosis and survival caspase cascade in plasma of Alzheimer’s patients are known for the prediction of disease (Bhatnagar et al., 2014). An analysis presented by Tan et al. (2014) showed that miRNAs are involved in the processes in pathogenesis associated with AD: amyloid-β accumulation, toxicity associated with tau proteins, inflammation, as well as neuronal cell death (Tan et al., 2014). In AD patients, activation, as well as inhibition of expression of *miR-9*, was found, which is enriched in the brain (Jin et al., 2013). *miR-133b* was downregulated in the midbrain of PD patients (Kim et al., 2007) as well as in mouse models of PD (Harraz et al., 2011; Filatova et al., 2012). However, studies are required to ascertain the presence of these miRNAs in circulation in these patients and their evaluation for potential biomarkers.

DNA methylation has a significant involvement in several neurodegenerative diseases (Al-Mahdawi et al., 2016). Increases and decrease in both 5mC and 5hmC at global levels have been identified in different diseases including AD (Al-Mahdawi et al., 2014). 5mC and 5hmC are identified as potential epigenetic markers in various neurodegenerative diseases both at global and locus-specific levels (Xiao et al., 2012; Sandi et al., 2013). However, further investigations are required for using 5mC and 5hmC as epigenetic biomarkers in cell-free circulating nucleic acids.

Previous studies have identified increased brain-derived ccfDNA in the serum of patients after traumatic brain injury (Lehmann-Werman et al., 2016). Rhomboid 5 Homolog 2 (*RHBDF2*) was found to be differentially methylated in the central nervous system (CNS) in Alzheimer’s disease (De Jager et al., 2014). In addition, a differentially methylated region located in the promoter–enhancer region of the *RHBDF2* gene was identified in amyotrophic lateral sclerosis (ALS) patients in ccfDNA in the plasma (Mendioroz et al., 2018). Thus, liquid biopsy may be applied to living patients as a source of potential epigenetic biomarkers for neurodegenerative disorders.

### Cell-Free miRNA in CSF and CNS Disorders

Levels of *miR-146a* and *miR-155*, the proinflammatory miRNAs, were found to be high in cerebrospinal fluid (CSF) of AD patients along with *miR-9* and *miR-125b* that are enriched in neurons (Alexandrov et al., 2012). Differential expression of *miR-15b* and *miR-21* was found in CSF from patients with primary CNS lymphoma, gliomas, and brain metastasis (Baraniskin et al., 2012). *miR-451* was detected in CSF microparticles after brain injury (Pigati et al., 2010). A list of ccfNAs studied in neurological diseases is provided in Table 4.

**TABLE 4:** Circulating cell-free nucleic acids (ccfNAs) in neurological diseases.

**Disease**	**Cell-free nucleic acids**	**Source**	**Potential diagnostic/prognostic biomarkers**	**References**
Traumatic brain injury (TBI)	Cell-free DNA	Brain	S100B, C-tau, NSE, and Hsp 70	Lorente, 2017
Alzheimer’s and Parkinson’s disease	Cell-free miRNA	Plasma	mir-34c	Bhatnagar et al., 2014
		Brain	miR-9	Jin et al., 2013
		Mid-brain	miR-133b	Kim et al., 2007
Friedreich’s ataxia (FRDA)	DNA methylation	Blood and buccal cells	5mC and 5hmC	Castaldo et al., 2008
Primary CNS lymphoma, gliomas, and brain metastasis	Cell-free miRNA	CSF	miR-146a, miR-155, miR-9, and miR-125b	Alexandrov et al., 2012
		CSF	miR-15b and miR-21	Baraniskin et al., 2012
		CSF microparticles	miR-451	Pigati et al., 2010
	ctDNA	CSF	Methylation of MGMT, p16/(INK4a), p73, and RARb	Balana et al., 2003; Weaver et al., 2006; Wakabayashi et al., 2009

**NSE, neuron-specific enolase; MGMT, O-6-methylguanine-DNA methyltransferase; p16/(INK4a), cyclin-dependent kinase inhibitor 2A, multiple tumor suppressor 1; RARb, retinoic acid receptor beta.**

## ccfNAs in Mitochondrial Diseases

In mitochondrial disorders, mitochondria fail to function properly and are not able to generate enough energy required for the body. These diseases are the chronic ones due to genetic causes, often inherited from the previous generation (Khan et al., 2015). Mitochondrial diseases can affect multiple organs of the body (Al-Enezi et al., 2008) and, in many conditions, can lead to secondary mitochondrial dysfunction like Lou Gehrig’s disease, diabetes, muscular dystrophy, Alzheimer’s disease, and cancer (Niyazov et al., 2016).

Commonly known mitochondrial diseases are mitochondrial myopathy, Leigh syndrome, Leber’s hereditary optic neuropathy (LHON), myoclonic epilepsy with ragged red fibers (MERRFs), myoneurogenic gastrointestinal encephalopathy (MNGIE), mitochondrial neurogastrointestinal encephalomyopathy (MNGIE), mitochondrial myopathy encephalomyopathy lactic acidosis stroke-like symptoms (MELAS), neuropathy ataxia retinitis pigmentosa and ptosis (NARP), and Friedreich’s ataxia (Khan et al., 2015).

### Potential of ccfNAs as Epigenetic Markers in Diagnosis, Progression, and Treatment of Mitochondrial Diseases

The diagnosis of mitochondrial diseases is difficult because it affects multiple organs, and hence, patients exhibit a variety of symptoms. Moreover, there is no single diagnostic or laboratory test that can accurately confirm a mitochondrial disease (Parikh et al., 2015). Therefore, investigation of ccfDNA or mtDNA in the plasma of patients independently can be a better biomarker.

Unlike nuclear DNA, mtDNA molecules are arranged in clusters, called nucleoids, which are tethered to the mitochondrial membrane and are devoid of histones (Holt, 2010). Earlier observations had suggested that mitochondria lacked the machinery required for DNA methylation. However, several strands of evidence later, including lower frequency of CG dinucleotides by bioinformatic analysis and modulation of mtDNA methylation in response to oxidative stress (Rebelo et al., 2009), suggested the other way. Additionally, DNA methylation of nuclear mitochondrial genes may play an important role in the understanding of mitochondrial disorders.

There has been some evidence that some types of histones do localize to the mitochondrial membrane, where mtDNA is tethered (Choi et al., 2011). In the nuclear DNA, histone modifications are important in transcriptional control that can be altered in diseases affecting nuclear-encoded mitochondrial proteins, of which Friedreich ataxia is an example. This disorder is caused by transcriptional silencing due to certain histone modifications of the *FXN* gene, which encodes a mitochondrial protein involved in the biosynthesis of iron–sulfur clusters (Rai et al., 2010). In one of the studies, total mtDNA in plasma was quantified and found to be high in Friedreich’s ataxia patients, which opened up its possible role as a blood-based biomarker (Swarup et al., 2011).

Serum microRNAs like *miR-1275*, *miR-149*, *miR-1*, *miR-133a*, *miR-133b*, *miR-145*, *miR-206*, *miR-208a*, *miR-208b*, *miR499*, and *miR-206* are reported in some of the studies to diagnose the muscle mitochondrial dysfunction (Cacchiarelli et al., 2011; Endo et al., 2013; Hu et al., 2014). In addition, in some metabolic diseases, where the mitochondria are not functioning properly like brown adipogenesis (Zhang et al., 2015), non-alcoholic fatty liver disease (Leti et al., 2015), and diabetes (Raffort et al., 2015), distinctive patterns of microRNA have been observed. In another study, mutations in cybrid cells identified the role of *microRNA-9/9*^∗^ pattern in different mitochondrial disorders, e.g., mitochondrial encephalomyopathy, lactic acidosis, and stroke-like episodes (MELAS) and myoclonic epilepsy with ragged-red fiber (MERRF) (Meseguer et al., 2015).

Hence, apart from the quantification of mtDNA in FRDA and serum microRNA profiles to assess mitochondrial myopathies, there is no available data on ccfNA epigenetic marker in mitochondrial diseases. Working on circulating mtDNA poses many challenges. mtDNA is highly polymorphic, which makes introducing targeted mutations into the mitochondria and generation of cellular or animal models of mitochondrial disorders quite challenging.

In addition, the gene panels required for the molecular diagnosis of mitochondrial disorders constitute an expensive approach. Therefore, the determination of epigenetic marks on ccfNA in plasma can have a diagnostic as well as prognostic potential in mitochondrial diseases. Thus, once the technical hurdles to study circulating mtDNA are taken care of by technology advancement, there is a strong motivation to explore the role of epigenetic mechanisms in mtDNA disease, as epigenetic factors may serve to explain the observed phenotypic heterogeneity, variable penetrance, and pronounced environmental triggers in this group of disorders.

## Artificial Intelligence/Machine Learning in ccfNA-Based Precision Medicine

There is a high impact of technologies such as high-performance computing as well as biological databases, like artificial intelligence (AI), machine learning (ML), and neural network, in the field of health care. Epigenetic data have traits like chemical and biological stability over time that make it open to ML (Xiong et al., 2015). Large-scale data-rich repositories such as The Cancer Genome Atlas (TCGA), BLUEPRINT, and the ENCODE association provide large amounts of samples to employ comprehensive, high-throughput statistical analysis of differentially methylated regions with biological relevance (Yuan et al., 2011; Munsell et al., 2015). Artificial intelligence and machine learning tools have especially found scope in cancer precision medicine by offering cancer patients personalized care. These methods help to decipher weak signals in the blood circulation at the early stages of cancer and provide a real-time assessment of cancer treatment. Nearly all datasets consist of DNA methylation profiles derived from peripheral blood, meaning that patients will only be required to provide a small blood sample. These can detect minute quantities of tumor DNA in blood and analyze their epigenetic marks for cancer monitoring.

Various programs have been generated to provide useful information for proper diagnosis. The *Graphite* is a bioconductor package to convert pathway topology to gene network (Sales et al., 2012). The *micrographite* package, for instance, provides a process to amalgamate mRNA and microRNA data via their association to canonical pathways (Calura et al., 2014). This approach has been beneficial in recognizing key microRNAs in primary myelofibrosis (Calura et al., 2016c), myeloma (Calura et al., 2016a), and ovarian cancer (Calura et al., 2016b). Another program, *Mergeomics* (multidimensional data integration to identify pathogenic perturbations to biological systems), combines data from epigenetic, genomic, and transcriptional association studies through a process of functional enrichment, which is used as the base for network construction; nevertheless, this tool has not been used in the context of cancer (Shu et al., 2016). Based on the multiomics data, *Netboost* is a network reconstruction method having statistical dependency and employs a commutable approach to lessen dimensionality. This system has been utilized for the categorization and survival study of acute myeloid leukemia data (Schlosser et al., 2020). Pair-wise relationships among various omics layers are identified by *AMARETTO*, which decides on cancer driver genes by taking into consideration frequently altered genes at the genome or epigenome level with functional consequences (Champion et al., 2018). Another tool *MAGIA* is used for the rebuilding of microRNA and transcription factor regulatory routes and has been employed for the scrutiny of expression and regulatory mechanisms in the NCI60 cell panel. As personalized genomic medicine pierces the age of “Big Data,” these would lead to uncovering of novel biomarkers on cancer indicators in the blood linked to particular disease states offered by machine learning algorithms. ML algorithms will assist with assessing the effects of various biomarkers concurrently and reveal top order interactions between biomarkers that would not be feasible to devise manually. A cancer genomics company, GRAIL, has launched a large-scale study, the Circulating Cell-Free Genome Atlas (CCGA), which uses machine learning to create a huge, representative library of cancer mutations and healthy mutations using data from ccfDNA and white blood cell genomes, to train their cancer screening algorithms (Cohn et al., 2019). With the availability of more data from clinical trials, this system can fine tune its algorithm to improve its diagnostic acumen.

Furthermore, artificial neural networks, which mimic the neurons of the brain, are functioning to interpret the data and provide the basis of machine learning. Disorders of neurodevelopment start early in childhood and have an impact on a diversity of functional domains as well as executive and cognitive function, social and language function, also behavior control, and motor function (Uddin et al., 2016; Thapar et al., 2017). Various other diagnoses include autism spectrum disorder (ASD) (Jiang et al., 2013), intellectual disability (ID) (Maulik et al., 2011), attention-deficit hyperactivity disorder (ADHD) (Lionel et al., 2011), and movement disorders (Huisman-Van Dijk et al., 2016). Consequently, the initiation of technologies dependent on transcriptome sequencing led to the foundation of the Allen developmental human brain atlas, profiling of the non-coding elements in the human genome by ENCODE database and the Human Cell Atlas (Kang et al., 2011; Regev et al., 2017). Recently, AI approaches have revealed by far reasonable success in neurodegenerative diseases (NDDs) by improving genetic diagnostics. The implementation of the Human Splicing Code is one of the first ML algorithms that demonstrate persuasive evidence of correctly categorizing disease-causing variants as well as those that are intronic. This process applies a Bayesian ML algorithm and has been illustrated in spinal muscular atrophy and pathogenic missense variants in ASD (Zhou and Troyanskaya, 2015). AI approaches are vital to explain the hidden arrangement in phenotype and genetic heterogeneity. NDDs are characterized by both phenotypic and genetic heterogeneity. For instance, cognitive function is impacted by 15q13.3 microdeletion syndrome and is found to be linked with heterogenous phenotypes that include speech delay (16%), epilepsy/seizure (57%), and ASD (11%) (Lowther et al., 2015). Although, biology enlightens this nosological evolution; however, more stress needs to be given on using AI approaches on major-scale datasets to authenticate or confront existing categorization paradigms.

## Conclusion and Future Perspective

**FIGURE 1:**
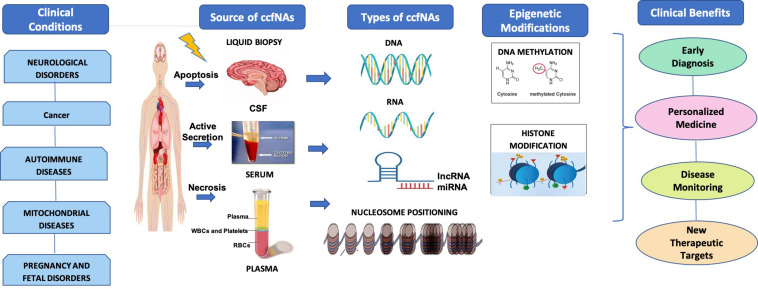
Under various clinical conditions such as neurological disorder, cancer, autoimmune diseases, mitochondrial diseases, and pregnancy and fetal disorders, circulating cell-free nucleic acids (ccfNAs) are released into body fluids like serum, plasma, and cerebrospinal fluids by apoptosis and necrosis. ccfNAs are of various types. The important ones are DNA, RNA, long non-coding RNAs (lncRNAs), and microRNA (miRNA) and have been observed to have disease-specific epigenetic modifications. These act as diagnostic and prognostic markers as well as therapeutic targets providing significant clinical benefits.

The utility of epigenetic alterations in ccfNA in various diseases as diagnostic and prognostic markers as well as therapeutic targets has been summarized in Figure 1. Liquid biopsy is fast replacing the invasive methods for the diagnosis and prognosis of various diseases. This method offers the possibility to separate and identify ccfNAs for their utility for screening, diagnostic, prognosis, or for selecting therapeutic options. The different types of ccfNAs being evaluated are ccfDNA, ccfRNA, ccfmtDNA, ccfmiRNA, ccflncRNA, etc. With the development of newer technologies for isolation of small amounts of ccfNAs and detection of the specific signatures on these, ccfDNA are already in clinical practice for a few diseases. Furthermore, recent advances in the field have shifted the focus from determining the quantity, SNPs, mutations of the ccfNAs to analyzing the epigenetic signatures like methylated sequences and nucleosome positioning, which are specific to the particular clinical condition. The epigenetic markers on the ccfNAs are widely being explored to further advance the field of personalized medicine. However, the genetic or epigenetic markers related to ccfNAs have paved the way in clinical practice mostly in cancer and prenatal screening only. For various other diseases like neurological, autoimmune, and mitochondrial diseases, there are limited data, most of which are limited to research findings only. There is a need to have a comprehensive data analysis of the epigenetic markers in ccfNAs in different physiological and pathological conditions and further testing of the selected markers in large population-based studies and disease cohorts. The field of epigenetic markers in ccfNAs holds tremendous potential in the field of precision medicine.

## Author Contributions

All authors listed have made a substantial, direct and intellectual contribution to the work, and approved it for publication.

## Conflict of Interest

The authors declare that the research was conducted in the absence of any commercial or financial relationships that could be construed as a potential conflict of interest.

## Abbreviations

5hmC: 5-hydroxy methyl cytosine
ccfDNAs: circulating cell-free deoxyribonucleic acids
ccf-fetal-NAs: circulating cell-free fetal nucleic acids
ccfmiRNAs: circulating cell-free miRNAs
ccfNAs: circulating cell-free nucleic acids
ccfRNAs: circulating cell-free ribonucleic acids
mtDNA: mitochondrial DNA.
